# Multimorbidity research in dementia: it’s time to shift to a person-centred approach

**DOI:** 10.3389/frdem.2026.1791795

**Published:** 2026-04-22

**Authors:** Subhashisa Swain, Kumud Kantilal, Vidyani Suryadevara, Lucy E. Stirland

**Affiliations:** 1 Nuffield Department of Primary Care Health Sciences, University of Oxford, Oxford, United Kingdom; 2 School of Medicine, Keele University, Newcastle-Under-Lyme, United Kingdom; 3 Research Department of Primary Care and Population Health, University College London, London, United Kingdom; 4 Department of Genetics, Stanford School of Medicine, Stanford University, Stanford, CA, United States; 5 Institute for Neuroscience and Cardiovascular Research, University of Edinburgh, Edinburgh, United Kingdom; 6 Global Brain Health Institute, University of California San Francisco, San Francisco, CA, United States

**Keywords:** dementia, multimorbidity, patient care, person-centered, primary care

## Abstract

People with dementia often live with several long-term conditions (known as multimorbidity). These conditions interact with dementia in complex ways and influence symptoms, care needs, and quality of life. Current researches are primarily divided into quantitative or qualitative methods without complementing each other. Most data driven research usually counts diagnoses or groups diseases. Qualitative evidence shows the need, and how daily routines, family roles, and personal goals affect care. These methods do not explain how people manage other long-term conditions alongside dementia. As a result, research findings do not always support care that centres on what matters to individuals. This paper proposes a shift in research on multimorbidity in people with dementia. Unlike other conditions, dementia is often diagnosed in later stage of life, with high burden of multimorbidity. We argue that data base research should focus on symptoms, function, treatment workload, and social context, which could shape how people live with dementia and multimorbidity. It can also inform the transitions between home, hospital, community services, and long-term care. These insights can guide the design of interventions that fit real situations. For evidence generation on symptom-based measures, functional assessments, and patient-reported outcomes, mixed methods studies can show how context, mechanisms, and outcomes interact. We offer a research agenda that places the person at the centre and what works for the patient going beyond the simple disease burden. This agenda supports the development of care that is feasible, acceptable, and meaningful. Without this shift, multimorbidity research may not improve everyday dementia care.

## Background

Dementia rarely occurs in isolation. Most individuals live with multiple long-term conditions (MLTCs), which create complex care needs influencing disease trajectories (Stirland et al., 2025). The presence of two or more long-term conditions (multimorbidity) (van den Akker et al., 1996) has emerged as a strong and dose-dependent risk factor for dementia (Shepherd et al., 2025). The interaction between dementia and multimorbidity is associated with accelerated cognitive and functional decline, increased healthcare utilization, impaired quality of life, and higher mortality risk (Browne et al., 2017; Melis et al., 2013). In the past 10 years, there has been a global surge in multimorbidity research, including on its relationship with dementia (Ahmed et al., 2020). However, research on multimorbidity and dementia varies widely in terms of concepts, definitions, purpose and implementation. Contemporary research in dementia and multimorbidity is focused on disease clustering, life-course accumulation, and risk stratification (Calvin et al., 2022; Patel et al., 2025). While these approaches provide valuable insights, they often fail to capture the lived experiences and priorities of those most affected. Taking a person-centred approach will extend our current understanding when undertaking research on dementia alongside multimorbidity. In their recently published paper, Stirland et al. (2025) rightly emphasised that multimorbidity is the norm rather than the exception among people living with dementia. An epidemiological study involving 6.5 million people in the UK showed that multimorbidity increased the risk of dementia two-fold (Shepherd et al., 2025). We agree that multimorbidity represents a critical determinant of dementia outcomes, and that single-disease paradigms are insufficient to address this complexity.

Dementia typically emerges after decades, at a stage when multimorbidity is usually already established (Calderón-Larrañaga et al., 2019). The clinical priorities after a diagnosis of dementia shift away from disease prevention towards managing symptoms, preserving function, and supporting quality of life. The key research challenge is therefore not simply to better quantify multimorbidity, but to understand the *experienced consequences* of multimorbidity, e.g., symptom burden, treatment burden (or workload), functional limitations, medication problems, and caregiver capacity. Research and care should be driven by symptoms, functional changes, and lived experiences rather than diagnostic counts. Classic multimorbidity approaches do not capture the fluctuating symptom burden, interaction effects between conditions, or the trade-offs that patient’s face when multiple treatments compete for attention and capacity. Systems designed around single diseases can struggle to support meaningful outcomes such as comfort, function, social participation, and caregiver capacity (Lynch et al., 2022), and they fall short in guiding transitions across care settings (home, hospital, long-term care) where priorities shift, and vulnerabilities intensify. These consequences shape care needs and transitions across the dementia trajectory along with multimorbidity burden. Without this shift in focus, multimorbidity research is unlikely to generate insights that meaningfully inform person-centred dementia care or improve outcomes that matter most to people living with the condition.

### Current research in multimorbidity and dementia

Biomedical research on multimorbidity and dementia has accumulated a substantial evidence base documenting associations between the number of chronic conditions and risk of cognitive decline or dementia outcomes (Stirland et al., 2025). However, this body of research remains heavily medicalised and focused on diagnostic counts, disease clusters, and epidemiological risk stratification rather than on the lived experience and care priorities of individuals. Traditional multimorbidity research assumes that “more conditions” directly translates to worse outcomes. However, dementia itself is a progressive, complex aging phenotype in which multimorbidity accumulate over decades, often long before cognitive impairment emerges. The current paradigm risks reinforcing siloed clinical models and lacks insight into how symptom burden, functional limitations, and individual priorities shape trajectories in real-world care. Moreover, conventional multimorbidity metrics are poorly aligned with the principles of person-centred care.

Person-centred care is widely endorsed in clinical practice guidelines, yet its principles remain underrepresented in dementia research (Guan et al., 2025; Serbser-Koal et al., 2024). Studies rarely incorporate patient-reported outcomes that reflect quality of life, functional ability, and symptom burden (Chang et al., 2019). This omission limits the development of interventions that align with what matters most to individuals, i.e., autonomy, comfort, and dignity. Furthermore, evidence on operationalizing person-centred approaches within the context of multimorbidity is sparse, leaving a gap between theoretical frameworks and practical intervention development and implementation (Dunn et al., 2022). A focus on symptom-based multimorbidity constructs, patient-reported outcomes, and longitudinal functional trajectories could more directly inform care that is responsive to individual goals. The current data infrastructures and cohort studies are insufficiently configured to support this work. Capturing what matters most in people with dementia will require methodological innovation, integrating qualitative insights, real-world symptom data, and dynamic measures of function.

In current dementia and multimorbidity research, study designs frequently prioritise biomedical indicators such as number of long-term conditions, hospital use and mortality, with limited attention to how people living with dementia define a good life or experience care across conditions (Poitras et al., 2018; Skou et al., 2022). Skou et al. (2022) analysed how the current multimorbidity research often aggregates coexisting diseases to estimate risks of premature death, functional decline and healthcare utilisation, reinforcing guidelines. In their review paper Poitras et al. (2018) provided a contrasting evidence on how a person-centred approach would begin with what matters most to individuals and their families, maintaining identity, autonomy, relationships and everyday routines. Person-centred interventions in dementia increasingly foreground personalised activities. Kim and Park in their systematic review highlighted how the meaning of person-centred care shifted to improvements in quality of life, neuropsychiatric symptoms and care experiences, even in the presence of complex multimorbidity and important for PLwD (Kim and Park, 2017). The contrast illustrates the practical implications of the proposed change: from counting conditions and clinical events to co-creating care plans that integrate multimorbidity management.

Addressing this gap is critical. Without a research paradigm that integrates patient perspectives and contextual realities, interventions risk being misaligned with individual needs and may fail to improve quality of life. Embedding co-design and participatory methods throughout the research process, alongside outcome measures that combine clinical indicators with patient-reported data, can generate evidence that informs policy and practice. Such approaches are essential to ensure that research not only advances scientific understanding but also delivers meaningful benefits for people living with dementia and multimorbidity.

Furthermore, multimorbidity research in dementia does more than drive clinical management. Recent work explicitly argues that care must balance chronic disease control with the risks of over-treatment and align decisions with what matters most to the person and family (Fried et al., 2020). Reviews of comorbidity/multimorbidity management show that most interventions and studies remain focused on medication counts and monitoring rather than goals, values, or everyday experience, and decision-making components are rare (Smith et al., 2012). Primary care physicians themselves describe “relaxed guideline adherence,” shifting away from tight disease control and instead trying to avoid burdensome treatment and inappropriate drugs (Mackey et al., 2012). However, they lack time, support and clear guidance on how to practice whole person, rather than disease based, care in dementia (Vinay and Biller-Andorno, 2023). Novel multimorbidity-specific measures of quality of life, although not specific to dementia, should be embedded in future trial design (Sweeney et al., 2014).

Emerging models point to a different use of multimorbidity knowledge: comprehensive geriatric assessment and patient-centred prescribing practices explicitly start from care goals (survival, function, symptom relief), then deprescribe or simplify regimens, treating multimorbidity only insofar as they support those goals (Aggarwal et al., 2020; Salisbury et al., 2020). Conceptual work on multimorbidity therefore converges with dementia specific reviews in calling for person-centred, minimally disruptive care that prioritises symptoms, daily functioning and relationships, and uses multimorbidity research to inform wiser, often less intensive, medical treatment rather than automatic escalation (Calvin et al., 2022).

Current data infrastructures for studying multimorbidity in dementia are poorly aligned with person-centred care. Most available datasets prioritise diagnoses, service use, and mortality, reflecting health-system needs rather than lived experience. As a result, multimorbidity is operationalised as a count or cluster of conditions, obscuring the symptoms, functional limitations, and personal priorities that shape daily life and care decisions in dementia. This limitation is particularly consequential given that dementia typically arises in later life, when the clinical management may focus less on preventing disease accumulation than balancing comfort, function, autonomy, and caregiver capacity and wellbeing. Person-centred outcomes, such as, symptom burden, treatment burden, and what matters most to individuals and families are inconsistently captured, often proxied, or entirely absent from routine health data. Addressing this gap will require a deliberate shift in research focus, away from disease-centric multimorbidity constructs and towards longitudinal, symptom- and function-based approaches that can meaningfully inform care transitions and support people living with dementia across the course of the condition.

Qualitative research on person-centred dementia care in the context of multimorbidity highlights the complex interplay between individuals’ cognitive decline, co-existing long-term conditions, and the fragmented systems that attempt to support them. A growing body of qualitative meta-syntheses demonstrates that people living with dementia and multiple conditions often experience gaps in continuity, coordination, and communication within care systems that are not well-designed for multimorbidity (Górska et al., 2018; Liao et al., 2023; Prorok et al., 2013), additionally, research on care transitions (Groenvynck et al., 2025; Handley et al., 2025; Morton et al., 2021) and digital technologies (Köhler et al., 2024; Notley et al., 2025) in dementia and multimorbidity offer granular insight into how and why care succeeds or fails in specific contexts. These studies consistently highlight mechanisms such as autonomy, trust, relational continuity, treatment burden and technology fit and how these are contingent on contextual conditions, ranging from digital infrastructure and regulation to family caregiving patterns and service fragmentation, to drive outcomes. Yet these nuanced insights have rarely been leveraged to shape population-level analyses. Harnessing this rich qualitative evidence could catalyse a shift to a new paradigm in dementia and multimorbidity research; one that is person-centred and goal-oriented and where mechanism and contexts are integrated into study designs, analysis and interpretation.

### Future directions for person-centred dementia and multimorbidity research

Advancing the field will require adopting a methodological position in which qualitative evidence is seen as integral rather than supplementary to the development of theory, construction of measurement tools and the modelling applied to large-scale datasets. Below we propose two approaches: Retrospective and prospective integration.

#### Retrospective integration: using existing qualitative findings to interrogate population-level data

Retrospectively, qualitative and realist studies can be used to interrogate and reshape the assumptions embedded within big-data research. Realist evaluations illuminate context, mechanism, and outcome configurations, such as, how transitions destabilise care when relational continuity is weak (Groenvynck et al., 2025; Handley et al., 2025), or how digital monitoring improves security only if it aligns with personal goals and is accompanied by human support (Köhler et al., 2024; Notley et al., 2025). These mechanisms can be operationalised as units of analysis within large datasets, shifting quantitative work from correlation-seeking to mechanism testing.

Qualitative insights can guide towards: (1) theory-driven measurement construction, using proxies for person-centred constructs, e.g., treatment burden (polypharmacy, treatment/care complexity), fragmentation (transition frequency and sequencing), or digital acceptability (patterns of telemonitoring alerts), (2) contextual stratification, ensuring that analyses reflect nuanced effects derived from realist methods, i.e., “for whom and under what circumstances” such as living alone, socioeconomic status, dementia stage, and (3) interpretive validity, allowing big-data findings to be interpreted through the lived experience of dementia and multimorbidity rather than through disease-centric assumptions. Our proposed approach reframes existing datasets as fertile ground for scaling and testing qualitative theory, not simply generating descriptive epidemiology.

#### Prospective integration: mixed-methods design where qualitative and quantitative findings iteratively inform one another

Future approaches should build purposeful mixed-methods designs where qualitative and quantitative streams are interconnected from the outset. We propose practical mixed-method integration strategies and combinations that could advance the science, thereby leveraging the full potential of qualitative insights and quantitative epidemiological, biomedical and data-driven enquiries.

*Qualitative to quantitative* to shape study designs. Participatory and co-design approaches with people with dementia and carers are used to define research questions, contexts, mechanism and outcomes that matter, e.g., autonomy, care continuity, minimally disruptive care. These could inform instrument development, e.g., measurement of treatment burden, priorities, and technology fit, surveys and data capture specifications, ensuring that person-centred constructs are measured at scale.

*Quantitative to qualitative* for seeking explanation. Population-level methods, e.g., multimorbidity modelling, cluster analysis, transition evaluation or home monitoring, could identify patterns and inequities that warrant deeper understanding. Targeted ethnography, realist interviewing and dyadic case studies could then explain why patterns emerge, refine programme theories and identify areas for improvement.

*Qualitative to quantitative* iterative cycles. Longitudinal mixed-methods cohorts can integrate remote monitoring streams, electronic health record linkage, and qualitative longitudinal interviews to capture the dynamic evolution of needs and preferences. Iteration will allow instruments and interventions to be adapted as abilities, goals, and contexts change, aligning with minimally disruptive, goal-oriented care.

This approach repositions qualitative inquiry as equally important to quantitative methods and will facilitate deeper understanding and do justice to the complexity of managing dementia and multimorbidity. Blending realist, ethnographic and qualitative research with population-level quantitative studies enables a shift in dementia and multimorbidity research. Retrospectively, qualitative mechanisms and contexts can be translated into testable constructs and stratifications within existing datasets, improving model validity and interpretation. Prospectively, iterative mixed-methods design ensure that qualitative and quantitative findings continually shape one another, producing measures, analyses and implementations that are person-centred. This integrated approach re-anchors the field’s values, re-states its causal assumptions and modernises its methods thereby advancing a research paradigm that is scientifically rigorous and attuned to the realities of living with dementia and multimorbidity.

**Figure 1:**
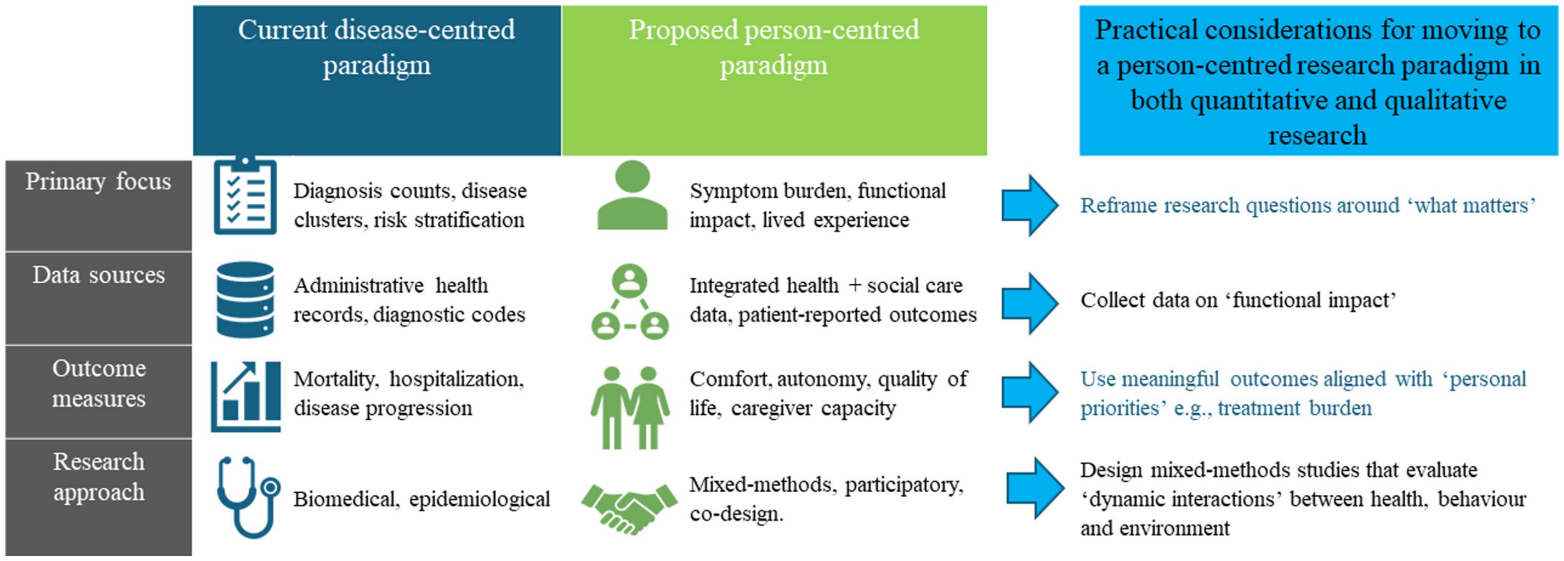
Shifting from disease-centric to person-centred paradigms in dementia research.

Future research on multimorbidity and dementia should move decisively beyond diagnosis-based frameworks towards approaches that capture symptom burden, functional impact, and *what matters* most to people living with dementia and their carers. Multimorbidity needs to be contextualized to the dementia population. This will require the routine integration of patient and caregiver reported outcomes into longitudinal studies, alongside traditional clinical and administrative data, to better reflect lived experience and evolving priorities over time. It will also align with, and inform, existing care provided by geriatricians and other specialist-generalist clinicians. We have suggested a framework for data that can enable patient-centred research in Figure 1. There is a need to identify or integrate the right data source and methods for the dementia population. Greater emphasis should be placed on studying care transitions as dynamic processes shaped by symptom trajectories, treatment burden, and caregiver capacity, rather than as isolated events. Achieving this shift will depend on methodological innovation, including improved use of proxy reporting, linkage of health and social care datasets, and development of ethically robust approaches to data collection in populations with cognitive impairment. Without such reorientation, multimorbidity research will continue to describe disease burden without meaningfully informing person-centred dementia care or improving outcomes across the later stages of life (Box 1).

BOX 1 **Future research agenda**

To advance person-centred multimorbidity research in dementia, future work should:

*Redefine multimorbidity constructs*

Move beyond diagnosis counts to symptom- and function-based measures that better reflect lived experience in dementia.

*Embed patient- and caregiver-reported outcomes*

Routinely collect longitudinal symptom burden, quality of life, treatment burden, and caregiver perspectives, using tools adapted for cognitive impairment.

*Link health and social care data*

Integrate administrative health records with social care, long-term care, and palliative care datasets to contextualise transitions and outcomes.

*Study transitions as experiences, not events*

Examine how symptom burden, priorities, and caregiver capacity shape transitions between care settings, rather than focusing solely on transition rates.

*Leverage technology to develop personalized approaches*

Explore the use of AI tools to develop patient-centric personalized approaches to improve the quality of life during dementia.

*Develop ethical and methodological innovations*

Address challenges in consent, proxy reporting, and fluctuating capacity through inclusive study designs and mixed methods approaches.

*Align outcomes with care goals*

Prioritise outcomes that matter to people living with dementia: comfort, function, dignity, and continuity, over disease-specific endpoints.

## Conclusion

To achieve workable, humane, and equitable dementia care in the presence of multimorbidity, research must study the world as people live in it. Person-centred research can reveal the unseen complexities, competing risks, and moral negotiations that structure everyday care. It provides the concepts and the language to design interventions that fit everyday life. The next phase is to ensure research truly fits with people’s needs and that interventions are acceptable, feasible, and have meaning. Without a person-centred approach, research may produce theoretically sound solutions that lack feasibility in real world care.

## Data Availability

The original contributions presented in the study are included in the article/supplementary material, further inquiries can be directed to the corresponding author/s.

## References

[ref1] Aggarwal P. Woolford S. J. Patel H. P. (2020). Multi-morbidity and polypharmacy in older people: challenges and opportunities for clinical practice. Geriatrics 5:85. doi: 10.3390/geriatrics5040085, 33126470 PMC7709573

[ref2] Ahmed M. A. A. Almirall J. Ngangue P. Poitras M.-E. Fortin M. (2020). A bibliometric analysis of multimorbidity from 2005 to 2019. J. Comorb. 10:2235042X2096528. doi: 10.1177/2235042X20965283, 33110764 PMC7557650

[ref3] Browne J. Edwards D. A. Rhodes K. M. Brimicombe D. J. Payne R. A. (2017). Association of comorbidity and health service usage among patients with dementia in the UK: a population-based study. BMJ Open 7:e012546. doi: 10.1136/bmjopen-2016-012546 PMC5353300 28279992

[ref4] Calderón-Larrañaga A. Vetrano D. L. Ferrucci L. Mercer S. W. Marengoni A. Onder G. . (2019). Multimorbidity and functional impairment–bidirectional interplay, synergistic effects and common pathways. J. Intern. Med. 285, 255–271. doi: 10.1111/joim.12843, 30357990 PMC6446236

[ref5] Calvin C. M. Conroy M. C. Moore S. F. Kuźma E. Littlejohns T. J. (2022). Association of Multimorbidity, disease clusters, and modification by genetic factors with risk of dementia. JAMA Netw. Open 5:e2232124. doi: 10.1001/jamanetworkopen.2022.32124, 36125811 PMC9490497

[ref6] Chang E. M. Gillespie E. F. Shaverdian N. (2019). Truthfulness in patient-reported outcomes: factors affecting patients’ responses and impact on data quality. Patient Relat. Outcome Meas. 10, 171–186. doi: 10.2147/PROM.S178344, 31354371 PMC6573779

[ref7] Dunn R. Clayton E. Wolverson E. Hilton A. (2022). Conceptualising comorbidity and multimorbidity in dementia: a scoping review and syndemic framework. J. Multimorb. Comorb. 12:263355652211284. doi: 10.1177/26335565221128432, 36187908 PMC9520180

[ref8] Fried T. R. Street R. L. Cohen A. B. (2020). Chronic disease decision making and ‘What Matters Most’. J. Am. Geriatr. Soc. 68, 474–477. doi: 10.1111/jgs.16371, 32043559 PMC7197748

[ref9] Górska S. Forsyth K. Maciver D. (2018). Living with dementia: a meta-synthesis of qualitative research on the lived experience. The Gerontologist 58, e180–e196. doi: 10.1093/geront/gnw195, 28069886 PMC5946830

[ref10] Groenvynck L. de Boer B. Beaulen A. de Vries E. Hamers J. P. H. van Achterberg T. . (2025). The real-time experiences of older people with dementia, informal caregivers and professional caregivers during the transition from home to a nursing home: multiple longitudinal cases. BMC Health Serv. Res. 25:1134. doi: 10.1186/s12913-025-13113-w, 40859260 PMC12382010

[ref11] Guan X. Duan A. -M. Xin G. Oyebode J. Liu Y. (2025). Barriers and facilitators to implementing person-centred dementia care in long-term care facilities in Western and Asian countries: a scoping review. Front. Psych. 15:1523501. doi: 10.3389/fpsyt.2024.1523501, 39876992 PMC11772481

[ref12] Handley M. Windle G. Mathie E. Greco H.-A. Underwood B. Surr C. . (2025). Living with dementia and other long-term conditions: what works for patient-caregiver dyads? A realist review. Aging Ment. Health 29, 1376–1386. doi: 10.1080/13607863.2025.2478168, 40176559

[ref13] Kim S. K. Park M. (2017). Effectiveness of person-centered care on people with dementia: a systematic review and meta-analysis. Clin. Interv. Aging 12, 381–397. doi: 10.2147/CIA.S117637, 28255234 PMC5322939

[ref14] Köhler S. Perry J. Biernetzky O. A. Kirste T. Teipel S. J. (2024). Ethics, design, and implementation criteria of digital assistive technologies for people with dementia from a multiple stakeholder perspective: a qualitative study. BMC Med. Ethics 25:84. doi: 10.1186/s12910-024-01080-6, 39068472 PMC11282641

[ref16] Liao L. Feng M. You Y. Chen Y. Guan C. Liu Y. (2023). Experiences of older people, healthcare providers and caregivers on implementing person-centered care for community-dwelling older people: a systematic review and qualitative meta-synthesis. BMC Geriatr. 23:207. doi: 10.1186/s12877-023-03915-0, 37003992 PMC10067217

[ref17] Lynch R. Hanckel B. Green J. (2022). The (failed) promise of multimorbidity: chronicity, biomedical categories, and public health. Crit. Public Health 32, 450–461. doi: 10.1080/09581596.2021.2017854, 38013883 PMC10461731

[ref18] Mackey K. Parchman M. L. Leykum L. Lanham H. Noel P. H. Zeber J. E. (2012). Impact of the chronic care model on medication adherence when patients perceive cost as a barrier. Prim. Care Diabetes 6, 137–142. doi: 10.1016/j.pcd.2011.12.004, 22264426 PMC3558316

[ref19] Melis R. J. F. Marengoni A. Rizzuto D. Teerenstra S. Kivipelto M. Angleman S. B. . (2013). The influence of multimorbidity on clinical progression of dementia in a population-based cohort. PLoS One 8:e84014. doi: 10.1371/journal.pone.0084014, 24386324 PMC3875493

[ref20] Morton T. Wong G. Atkinson T. Brooker D. (2021). Sustaining community-based interventions for people affected by dementia long term: the SCI-Dem realist review. BMJ Open 11:e047789. doi: 10.1136/bmjopen-2020-047789 PMC8264885 34233990

[ref21] Notley N. Worthy P. Peters M. E. Shaw J. Nelson A. Frost D. . (2025). Encountering technology: a qualitative exploration of the technology experiences of people living with dementia and their care partners to inform knowledge, practice and technology design. Int. Rev. Psychiatry 1–14. doi: 10.1080/09540261.2025.2533898

[ref22] Patel R. Gillis G. Mackay C. E. Griffanti L. Wang C. Ebmeier K. P. . (2025). The lifetime accumulation of multimorbidity and its influence on dementia risk: a UK biobank study. Brain Commun. 7:fcaf222. doi: 10.1093/braincomms/fcaf222, 40672935 PMC12266833

[ref23] Poitras M.-E. Maltais M.-E. Bestard-Denommé L. Stewart M. Fortin M. (2018). What are the effective elements in patient-centered and multimorbidity care? A scoping review. BMC Health Serv. Res. 18:446. doi: 10.1186/s12913-018-3213-8, 29898713 PMC6001147

[ref24] Prorok J. C. Horgan S. Seitz D. P. (2013). Health care experiences of people with dementia and their caregivers: a meta-ethnographic analysis of qualitative studies. Can. Med. Assoc. J. 185, E669–E680. doi: 10.1503/cmaj.121795, 24003093 PMC3787191

[ref25] Salisbury C. Lay-Flurrie S. Bankhead C. R. Fuller A. Murphy M. Caddick B. . (2020). Measuring the complexity of general practice consultations: a Delphi and cross-sectional study in English primary care. Br. J. Gen. Pract. 71, e423–e431. doi: 10.3399/bjgp.2020.0486, 33824162 PMC8049201

[ref26] Serbser-Koal J. Rommerskirch-Manietta M. Purwins D. Roes M. (2024). Person-centredness in dementia care: an integrative review of theoretical approaches. BMJ Open 14:e085051. doi: 10.1136/bmjopen-2024-085051, 38951009 PMC11218012

[ref27] Shepherd H. Todd A. Sinclair D. R. Richardson C. L. Matthews F. E. Kingston A. (2025). The association between multiple long-term conditions and dementia: a UK cohort study. Alzheimers Dement. (Amst) 17:e70230. doi: 10.1002/dad2.70230, 41404483 PMC12703650

[ref28] Skou S. T. Mair F. S. Fortin M. Guthrie B. Nunes B. P. Miranda J. J. . (2022). Multimorbidity. Nat. Rev. Dis. Primers 8:48. doi: 10.1038/s41572-022-00376-4, 35835758 PMC7613517

[ref29] Smith S. M. Soubhi H. Fortin M. Hudon C. O’Dowd T. (2012). Managing patients with multimorbidity: systematic review of interventions in primary care and community settings. BMJ 345:e5205. doi: 10.1136/bmj.e5205, 22945950 PMC3432635

[ref15] Stirland L. E. Choate R. Zanwar P. P. Zhang P. Watermeyer T. J. Valletta M. . (2025). Multimorbidity in dementia: current perspectives and future challenges. Alzheimers Dement. 21:e70546. doi: 10.1002/alz.70546, 40755143 PMC12319240

[ref30] Sweeney J. McHugh S. Perry I. J. (2014). Implementation and evaluation of a clinical data management programme in a primary care centre. Ir. Med. J. 107, 323–326, 25556259

[ref31] van den Akker M. Buntinx F. Knottnerus J. A. (1996). Comorbidity or multimorbidity: what’s in a name? A review of literature. Eur. J. Gen. Pract. 2, 65–70. doi: 10.3109/13814789609162146

[ref32] Vinay R. Biller-Andorno N. (2023). A critical analysis of national dementia care guidances. Health Policy 130:104736. doi: 10.1016/j.healthpol.2023.104736, 36796180

